# The Impacts of* SLC22A1* rs594709 and* SLC47A1* rs2289669 Polymorphisms on Metformin Therapeutic Efficacy in Chinese Type 2 Diabetes Patients

**DOI:** 10.1155/2016/4350712

**Published:** 2016-02-10

**Authors:** Di Xiao, Yu Guo, Xi Li, Ji-Ye Yin, Wei Zheng, Xin-Wen Qiu, Ling Xiao, Rang-Ru Liu, Sai-Ying Wang, Wei-Jing Gong, Hong-Hao Zhou, Zhao-Qian Liu

**Affiliations:** ^1^Department of Clinical Pharmacology, Xiangya Hospital, Central South University, Changsha 410008, China; ^2^Institute of Clinical Pharmacology, Central South University and Hunan Key Laboratory of Pharmacogenetics, Changsha 410078, China; ^3^Hunan Province Cooperation Innovation Center for Molecular Target New Drug Study, Hengyang 421001, China; ^4^Changsha Medical University Teaching Hospital, The People's Hospital of Liuyang, Liuyang 410300, China

## Abstract

*Background*. We aimed to investigate the distributive characteristics of* SLC22A1* rs594709 and* SLC47A1* rs2289669 polymorphisms and their influence on metformin efficacy in Chinese T2DM patients.* Methods*. The distributions of* SLC22A1* rs594709 and* SLC47A1* rs2289669 polymorphisms were determined in 267 T2DM patients and 182 healthy subjects. Subsequently, 53 newly diagnosed patients who received metformin monotherapy were recruited to evaluate metformin efficacy.* Results*. No significant difference was found between T2DM patients and healthy subjects in* SLC22A1* rs594709 and* SLC47A1* rs2289669 allele frequencies and genotype frequencies. After metformin treatment,* SLC22A1* rs594709 GG genotype patients showed a higher increase in FINS (*p* = 0.015) and decrease in HOMA-IS (*p* = 0.001) and QUICKI (*p* = 0.002) than A allele carriers.* SLC47A1* rs2289669 GG genotype patients had a higher decrease in TChol (*p* = 0.030) and LDL-C (*p* = 0.049) than A allele carriers. Among* SLC22A1* rs594709 AA genotype, patients with* SLC47A1* rs2289669 AA genotype showed a higher decrease in FBG (*p* = 0.015), PINS (*p* = 0.041), and HOMA-IR (*p* = 0.014) than G allele carriers. However, among* SLC22A1* rs594709 G allele carriers,* SLC47A1* rs2289669 AA genotype patients showed a higher decrease in TChol (*p* = 0.013) than G allele carriers.* Conclusion*. Our data suggest that* SLC22A1* rs594709 and* SLC47A1* rs2289669 polymorphisms may influence metformin efficacy together in Chinese T2DM patients.

## 1. Introduction

Metformin is the most widely used oral antidiabetic drug, which could effectively lower blood glucose and reduce the risk of cardiovascular diseases [1]. The classical functional mechanism of metformin is to decrease the hepatic energy status by inhibiting the mitochondrial respiratory-chain complex 1, thus activating the adenosine 5′-monophosphate-activated protein kinase (AMPK), a cellular metabolic sensor, to finally lower the blood glucose level [2]. In recent years, further probable mechanisms involved in cAMP, the phosphorylation of Acc1 and Acc2, mitochondrial glycerophosphate dehydrogenase, and the activated duodenal AMPK were found to affect metformin's effect in different ways [3–6]. With its significant glycemic benefits and neutral effects on weight, metformin remains the recommended first-line oral therapy in the guidelines of the American Diabetes Association and European Association of the Study of Diabetes [7]. However, during its clinical usage, the individual differences and drug failure widely existed [8, 9].

Metformin is not metabolized* in vivo*. The gastrointestinal absorption, hepatic uptake, and renal excretion of metformin are mediated greatly by organic cation transporters (OCTs) and multidrug and toxin extrusion proteins (MATEs) [10]. It was widely reported that genetic variants in coding genes of transporters mentioned above played important roles in pharmacokinetics and pharmacodynamics of metformin [9, 11–13]. Among these,* SLC22A1* rs628031 (tagged to rs594709 in Asian) was reported to affect imatinib uptake in chronic myeloid leukemia (CML) cell line [14]. As we know, imatinib is also transported by OCT1 in the human organism.* SLC47A1* rs2289669 was reported to be associated with metformin excretion [15] as well as its therapeutic response [16, 17]. Besides, a gene-gene interaction between genetic variants in* SLC47A1* and* SLC22A1*,* SLC47A1* and* SLC22A2* had a significant influence on metformin's efficacy [18, 19]. It was found that there was a SNP-SNP interaction between* SLC47A1* rs2289669 and* SLC22A1* rs622342 [18]. However, the effect of* SLC22A1* rs594709 and* SLC47A1* rs2289669 polymorphisms and SNP-SNP interaction on metformin efficacy in Chinese T2DM patients has not been widely evaluated.

In this study, we aimed to investigate the distributive characteristics of* SLC22A1* rs594709 and* SLC47A1* rs2289669 between T2DM patients and healthy subjects and the influences of* SLC22A1* rs594709 and* SLC47A1* rs2289669 polymorphisms on metformin's therapeutic efficacy in glucose lowering, serum lipids, and insulin sensitivity. Moreover, we are interested in whether there is a SNP-SNP interaction between* SLC22A1* rs594709 and* SLC47A1* rs2289669 on metformin's therapeutic efficacy.

## 2. Material and Methods

### 2.1. Subjects

Distributive characteristics of* SLC22A1* rs594709 and* SLC47A1* rs2289669 polymorphisms were determined in 267 unrelated patients with T2DM and 182 healthy subjects. 267 T2DM patients (143 males and 124 females) were recruited from the Second Xiangya Hospital of Central South University, the People's Hospital of Liuyang, and the First Hospital of Changsha. 182 healthy subjects (86 males and 96 females) were recruited from Physical Examination Center of Xiangya Hospital of Central South University. T2DM was diagnosed according to DM diagnostic criteria of WHO 1999. Patients with type 1 diabetes, gestational diabetes, uncontrollable hypertension, severe liver, and kidney disease were not included in this study.

Furthermore, 53 of 267 T2DM patients who were newly diagnosed and drug naïve (31 males and 22 females; average age 49 years, range 29–73 years) were enrolled to evaluate the effect of metformin among different* SLC22A1* rs594709 and* SLC47A1* rs2289669 genotype individuals. Newly diagnosed type 2 diabetes was defined as being diagnosed with T2DM within 6 months before enrollment. All patients had not been treated with any insulin secretagogue and received metformin monotherapy for 3 consecutive months. Metformin doses ranged from 1000 mg/day to 2000 mg/day among individuals. The study was approved by the Ethics Committee of Xiangya School of Medicine, Central South University (Changsha, Hunan, China) (CTXY-110002-5), and was performed in accordance with the Helsinki Declaration. Written informed consent was provided by all subjects.

### 2.2. Clinical Laboratory Tests

Detailed clinical information was collected from each patient. Body mass index (BMI), fasting blood glucose (FBG), postprandial blood glucose (PBG), fasting insulin (FINS), postprandial insulin (PINS), glycosylated haemoglobin (HbA1c), total cholesterol (TChol), triglycerides (TG), low-density lipoprotein cholesterol (LDL-C), and high-density lipoprotein cholesterol (HDL-C) were evaluated before and after metformin treatment as previously described [20, 21]. Homeostasis model assessment of insulin resistance (HOMA-IR), homeostasis model assessment of insulin sensitivity (HOMA-IS), homeostasis model assessment of *β*-cell function (HOMA-B), and quantitative insulin sensitivity check index (QUICKI) were calculated according to previous formulas [22, 23].

### 2.3. DNA Sampling and Genotyping

Genomic DNA was isolated from peripheral blood leukocytes using Promega DNA purification kit (Promega, Wisconsin, USA) according to the manufacturer's protocol and stored at −20°C until use. Genotyping was done using direct sequencing. The primers used to amplify* SLC22A1* rs594709 polymorphism were 5′GGCGCTTCCCACACTCAT3′ (sense) and 5′GAGGAAAGCTCCACATGTAACC3′ (antisense), and the primers for* SLC47A1* rs2289669 polymorphism were 5′CCTGACCTCTGTCCCTATT3′ (sense) and 5′GACTTCCGTGCCTGCT3′ (antisense). The PCR products were then sequenced using ABI 3700 automated sequencer (ABI, Foster City, CA, USA).

### 2.4. Statistical Analysis

Independent-samples* t*-test was used to compare baseline characteristics in T2DM patients and healthy subjects. The observed genotype frequencies in patients and controls compared with expected frequencies under Hardy-Weinberg equilibrium were evaluated by *χ* ^2^ test. Comparisons of frequencies of genotypes and alleles were also analyzed by *χ* ^2^ test.

We evaluated associations between each SNP and the change of clinical index before and after metformin therapy using multivariate linear regression by adjusting for sex, age, and BMI in additive model, recessive model, and dominant model, respectively. Besides, the analysis for the* SLC22A1 rs594709* genotype was stratified for the* SLC47A1* rs2289669 genotype and vice versa. Multivariate linear regression was used to analyze the interaction between* SLC22A1* rs594709 and* SLC47A1* rs2289669 genotype on metformin response in Chinese T2DM patients. All of the analyses were performed with SPSS software (version 16.0; SPSS, Chicago, Illinois, USA).

## 3. Results

### 3.1. Clinical Characteristics of Subjects

A total of 267 T2DM patients and 182 healthy subjects were enrolled to explore the allele frequencies and genotype frequencies of* SLC22A1* rs594709 and* SLC47A1* rs2289669 polymorphisms. The clinical characteristics of T2DM patients and healthy subjects were shown in Table 1. BMI (25.10 ± 2.74 kg/m^2^ versus 23.98 ± 2.94 kg/m^2^, *p* < 0.001), waist circumference (87.40 ± 8.56 cm versus 81.72 ± 9.52 cm, *p* < 0.001), waist-to-hip ratio (0.91 ± 0.06 versus 0.85 ± 0.07, *p* < 0.001), FBG (8.91 ± 3.40 mmol/L versus 5.11 ± 0.57 mmol/L, *p* < 0.001), and TG (3.12 ± 3.71 mmol/L versus 1.85 ± 6.82 mmol/L, *p* < 0.05) in T2DM patients were significantly higher than those of healthy subjects. Moreover, HDL-C level (1.32 ± 0.64 mmol/L versus 1.51 ± 0.45 mmol/L, *p* < 0.001) in T2DM patients was significantly lower than that in healthy subjects. No significant differences were found in hip circumference, TChol, and LDL-C level between two groups.

### 3.2. Genotype Analysis and Allelic Frequencies

The frequencies of the* SLC22A1* rs594709 and* SLC47A1* rs2289669 polymorphisms in T2DM patients and healthy subjects were shown in Table 2. Both SNPs were in agreement with Hardy-Weinberg equilibrium in each group (*p* > 0.05). Minor allele frequencies (MAFs) of* SLC22A1* rs594709 in T2DM group and healthy control were 26.78% and 28.57%, respectively. MAFs of* SLC47A1* rs2289669 were, respectively, 47.19% and 46.97% in T2DM patients and healthy subjects. No significant differences were found in allele frequencies (*p* = 0.555 for rs594709; *p* = 0.950 for rs2289669) and genotype frequencies (*p* = 0.927 for rs594709; *p* = 0.669 for rs2289669) between T2DM patients and healthy controls.

### 3.3. Influence of* SLC22A1* rs594709 and* SLC47A1* rs2289669 Polymorphisms on Therapeutic Efficacy of Metformin in T2DM Patients

53 newly diagnosed T2DM patients who had taken the first prescription of metformin for 3 consecutive months were enrolled to evaluate metformin efficacy. As shown in Table 3,* SLC22A1* rs594709 GG genotype patients had a higher increase in FINS (*p* = 0.015) and greater decrease in HOMA-IS (*p* = 0.001) and QUICKI (*p* = 0.002) than those of A allele carriers (Table 3). However,* SLC47A1* rs2289669 polymorphism was associated with changes in TChol and LDL-C level; patients carrying* SLC47A1* rs2289669 A allele had a relatively minor decrease in TChol (*p* = 0.030) and LDL-C (*p* = 0.049) level compared to GG genotype patients (Table 3).

Additionally, we found a significant SNP-SNP interaction between* SLC47A1* rs2289669 and* SLC22A1* rs594709, which affected the improvement of insulin resistance and blood lipid induced by metformin therapy. Among* SLC22A1* rs594709 AA genotypes, patients with* SLC47A1* rs2289669 AA genotype showed significantly higher decrease in FBG (*p* = 0.015), PINS (*p* = 0.041), and HOMA-IR (*p* = 0.014) than GA or GG genotypes (Table 4), while, among* SLC22A1* rs594709 G allele carriers, patients with* SLC47A1* rs2289669 AA genotype showed a greater decrease in TChol (*p* = 0.013) than GA or GG genotypes (Table 4).

## 4. Discussion

In the current study, we evaluated the distributive characteristics of* SLC22A1* rs594709 and* SLC47A1* rs2289669 polymorphisms and the separate as well as combined influence of their polymorphisms on metformin's effect in Chinese T2DM patients. Our results showed that there were no significant differences in* SLC22A1* rs594709 and* SLC47A1* rs2289669 allele frequencies and genotype frequencies between T2DM patients and healthy subjects.* SLC22A1* rs594709 A allele carriers showed a better metformin efficacy in insulin sensitivity compared with GG genotype patients.* SLC47A1* rs2289669 GG genotype patients showed a better efficacy in improving blood lipid. Furthermore, we found that there was a significant interaction between* SLC47A1* rs2289669 and* SLC22A1* rs594709, which might function together in metformin efficacy.

OCTs mediate the interactions between cells and their environment and are involved in transporting a broad spectrum of endogenous substrates and the detoxification of xenobiotics [24]. Nutrient transporters, including OCT1 and MATE1, could affect nutrient homeostasis through different ways [25]. Polymorphisms in OCT1 or MATE1 have been correlated with the susceptibility of type 2 diabetes, diabetic nephropathy, hypertension, and primary biliary cirrhosis [26–28].* SLC47A1* rs2453583 and* SLC22A1* rs651154 have been reported to be nominally associated with diabetes incidence in the DPP (Diabetes Prevention Program) [26]. Therefore, we investigated the distributive characteristics of the two SNPs that we are concerned with, namely,* SLC22A1* rs594709 and* SLC47A1* rs2289669, in T2DM patients and healthy subjects. However, no statistical difference was found between two groups.

Human* SLC22A1* gene is highly polymorphic, and several polymorphisms have been described to affect metformin responses [29, 30], including polymorphisms located in the intron regions [31].* SLC22A1* rs594709 locates in intron 4. Pairwise linkage disequilibrium (LD) analysis indicated that there were strong LDs between rs594709 and rs2282143, rs628031, rs3798168, and rs683369. Polymorphisms mentioned above could more or less affect the metabolizing and/or efficacy of OCT1 substrates.* SLC22A1* rs2282143 variation led to a decrease of transport activity, but not completely abolished [32].* SLC22A1* rs628031 (Met408Val) was identified in multiethnic groups worldwide [33] and has been characterized to have normal metformin uptake* in vitro* [9, 11]. However, it has been recognized as a weak positive predictor of metformin response in clinical study [8, 34, 35], though this genetic variant did not alter protein expression [34, 36]. Recent studies reported that* SLC22A1* rs3798168, rs683369, and rs628031 could affect the response to imatinib therapy in chronic myeloid leukemia (CML) patients [37, 38]. As* SLC22A1* rs594709 is the tagger SNP of rs3798168, rs683369, and rs628031 (*r* ^2^ > 0.8), it is possible that the structural changes in* SLC22A1* caused by any of these polymorphisms or even a combination effect of them may affect its transport ability of metformin.

Previously, Caucasians population-based study in diabetic patients revealed that rs2289669 G/A in* SLC47A1* gene was associated with the therapeutic efficacy of metformin [16, 17]. The decrease in HbA1c was greater in AA homozygotes compared with the GA + GG genotype patients after metformin treatment. The effect of* SLC47A1* rs2289669 polymorphism on pharmacodynamics (*n* = 202) and pharmacokinetics (*n* = 28) of metformin was also evaluated in Chinese patients and showed that AA genotype patients had a better glucose lowering effect after one-year follow-up. As for pharmacokinetics parameters, AA genotype patients had higher AUC_12h_ and lower renal clearance and renal clearance by secretion [15]. Thus, it is possible that the effect of the rs2289669 polymorphism on metformin glucose lowering efficacy was caused partly or completely by a reduced renal excretion and increased metformin plasma levels [39]. However, in our study, we did not find* SLC47A1* rs2289669 polymorphism was correlated with metformin glucose lowering effect but was associated with the reduction of TChol and LDL-C. This result was similar to long-term metformin and sulphonylurea combination therapy study carried out in Slovenia [40]. Their results suggested that* SLC47A1* rs2289669 genotype was not associated with HbA1c levels but significantly associated with cholesterol levels in metformin-treated T2DM patients even after adjustment for renal function. In contrast to the previous studies, the sample size, period of metformin treatment, and different races were all possible reasons leading to different results. Replication studies in a relatively large population in a multi-ethnic diabetic cohort are needed to confirm* SLC47A1* rs2289669 polymorphism effect on metformin efficacy both in glycemic control and in lipid profiles.

Furthermore, we found that there was an interaction between* SLC47A1* rs2289669 and* SLC22A1* rs594709, which affected the blood glucose, insulin level, insulin resistance improvement, and blood lipid after metformin treatment. Among* SLC22A1* rs594709 AA genotype patients,* SLC47A1* rs2289669 AA genotype patients showed a better glycemic control compared with G allele carriers, while, among* SLC22A1* rs594709 G allele carriers, patients with* SLC47A1* rs2289669 AA genotype showed a better metformin efficacy in blood lipid than GA or GG genotypes. Interaction between* SLC22A1* and* SLC47A1* polymorphisms and metformin response had been reported previously [18]; the effect of the* SLC47A1* rs2289669 polymorphism on the decrease of HbA1c by metformin was larger in incident users with the* SLC22A1* rs622342 CC genotype than in incident users with the AA or AC genotype patients. Both of the rs622342 and rs594709 are located in intron regions of* SLC22A1* gene. The interaction is possibly due to the genetic mutations in* SLC22A1* and* SLC47A1* altering the function of OCT1-mediated influx and MATE1-mediated efflux. As OCT1 transports metformin into the hepatocyte and MATE1 transports metformin out of the hepatocyte into the bile, altered OCT1 and MATE1 function may be able to influence the intracellular metformin concentrations and glucose lowering function of metformin. Further replication and mechanism studies are needed to illustrate the influences of* SLC22A1* and* SLC47A1* polymorphisms on metformin efficacy.

In summary, we found that* SLC22A1* 594709 and* SLC47A1* rs2289669 polymorphisms may interact to affect the therapeutic efficacy of metformin in Chinese T2DM patients. From our data, T2DM patients with AA genotype of both* SLC22A1* rs594709 and* SLC47A1* rs2289669 may get maximum glucose lowering benefit from metformin monotherapy. The* SLC22A1* and* SLC47A1* gene-gene interaction further suggests the complexity of metformin efficacy in T2DM patients.

## Figures and Tables

**Table 1:** The clinical characteristics of T2DM patients and healthy subjects.

Parameter	Healthy controls (*n* = 182)	T2DM patients (*n* = 267)	*p* value
Gender			
Male	86	143	
Female	96	124	0.189
Age (years)	49.04 ± 8.10	49.82 ± 10.11	0.370
BMI (kg/m^2^)	23.98 ± 2.94	25.10 ± 2.74	0.000^*∗∗∗*^
Waist circumference (cm)	81.72 ± 9.52	87.40 ± 8.56	0.000^*∗∗∗*^
Hip circumference (cm)	95.32 ± 6.46	96.49 ± 7.36	0.097
Waist-hip ratio	0.85 ± 0.07	0.91 ± 0.06	0.000^*∗∗∗*^
FBG (mmol/L)	5.11 ± 0.57	8.91 ± 3.40	0.000^*∗∗∗*^
TG (mmol/L)	1.85 ± 6.82	3.12 ± 3.71	0.025^*∗*^
TChol (mmol/L)	4.88 ± 0.97	5.05 ± 1.81	0.255
LDL-C (mmol/L)	2.82 ± 0.78	2.90 ± 1.01	0.388
HDL-C (mmol/L)	1.51 ± 0.45	1.32 ± 0.64	0.000^*∗∗∗*^

BMI: body mass index; FBG: fasting blood glucose; TG: triglycerides; TChol: total cholesterol; LDL-C: low-density lipoprotein cholesterol; HDL-C: high-density lipoprotein cholesterol. ^*∗*^ *p* < 0.05, ^*∗∗∗*^ *p* < 0.001.

**Table 2:** Comparisons of allelic frequencies of *SLC22A1* rs594709 and *SLC47A1* rs2289669 polymorphisms in T2DM patients and healthy subjects.

Genotype	T2DM patients (*n* = 267)	Healthy controls (*n* = 182)	*p* value
*SLC22A1* rs594709			
AA	146 (54.68%)	95 (52.20%)	0.927
GA	99 (37.08%)	70 (38.46%)	
GG	22 (8.24%)	17 (9.34%)	
Alleles			
A	391 (73.22%)	260 (71.43%)	0.555
G	143 (26.78%)	104 (28.57%)	
*SLC47A1* rs2289669			
AA	77 (28.84%)	49 (26.92%)	0.669
GA	128 (47.94%)	95 (52.20%)	
GG	62 (23.22%)	38 (20.88%)	
Alleles			
A	282 (52.81%)	193 (53.02%)	0.950
G	252 (47.19%)	171 (46.97%)	

**Table 3:** Comparisons of differential values (post-administration minus pre-administration) in T2DM patients with different *SLC22A1* rs594709 and *SLC47A1* rs2289669 genotypes before and after metformin treatment.

Parameter	*SLC22A1* rs594709		*p* value	*SLC47A1* rs2289669		*p* value
	AA/GA (*n* = 50)	GG (*n* = 3)		AA/GA (*n* = 37)	GG (*n* = 16)	
FBG (mmol/L)	−2.02 ± 2.84	−0.10 ± 0.62	0.112	−2.15 ± 3.11	−1.27 ± 1.72	0.668
PBG (mmol/L)	−5.28 ± 6.03	−1.73 ± 2.66	0.171	−5.61 ± 6.17	−3.84 ± 5.32	0.692
FINS (mU/L)	3.90 ± 5.40	11.35 ± 4.46	0.015^a^	4.07 ± 6.06	5.19 ± 4.47	0.501
PINS (mU/L)	−9.77 ± 25.58	−20.58 ± 22.72	0.259	−13.69 ± 29.73	−3.53 ± 6.81	0.457
HbA1c (%)	−2.83 ± 4.82	−0.60 ± 0.53	0.227	−2.89 ± 5.38	−2.21 ± 2.64	1.000
TG (mmol/L)	−0.84 ± 3.71	−0.74 ± 1.28	0.434	−0.72 ± 3.71	−1.10 ± 3.44	0.163
TChol (mmol/L)	−0.60 ± 1.46	0.05 ± 1.56	0.224	−0.39 ± 1.40	−0.96 ± 1.55	0.030^b^
LDL-C (mmol/L)	−0.14 ± 1.00	−0.09 ± 0.78	0.451	−0.08 ± 1.10	−0.28 ± 0.63	0.049^b^
HDL-C (mmol/L)	−0.03 ± 0.18	−0.05 ± 0.15	0.399	−0.02 ± 0.19	−0.08 ± 0.14	0.254
HOMA-IR	0.60 ± 2.87	3.06 ± 0.72	0.081	0.55 ± 3.27	1.31 ± 1.31	0.357
HOMA-IS	−0.003 ± 0.03	−0.08 ± 0.10	0.001^a^	−0.009 ± 0.05	−0.009 ± 0.02	0.382
HOMA-B	0.90 ± 1.70	0.96 ± 0.70	0.493	0.86 ± 1.48	1.01 ± 2.06	0.295
QUICKI	−0.03 ± 0.17	−0.43 ± 0.46	0.002^a^	−0.05 ± 0.25	−0.07 ± 0.10	0.446

BMI: body mass index; FBG: fasting blood glucose; PBG: postprandial blood glucose; FINS: fasting insulin; PINS: postprandial insulin; HbA1c: glycosylated haemoglobin; TG: triglycerides; TChol: total cholesterol; LDL-C: low-density lipoprotein cholesterol; HDL-C: high-density lipoprotein cholesterol; HOMA-IR: homeostasis model assessment of insulin resistance; HOMA-IS: homeostasis model assessment of insulin sensitivity; HOMA-B: homeostasis model assessment of *β*-cell function; QUICKI: quantitative insulin sensitivity check index.

^a^ *p* < 0.05 for comparison of GG genotype with GA/AA genotype of *SLC22A1* rs594709 polymorphism, adjusted for age, sex, and BMI, in recessive model.

^b^ *p* < 0.05 for comparison of GG genotype with GA/AA genotype of *SLC47A1* rs2289669 polymorphism, adjusted for age, sex, and BMI, in recessive model.

**Table 4:** The differential values (DV) of clinical characteristics per *SLC22A1* rs594709 and *SLC47A1 *rs2289669 genotypes.

*SLC47A1* rs2289669	*SLC22A1* rs594709 AA (*n* = 32)		*p* value	*SLC22A1* rs594709 GA/GG (*n* = 21)		*p* value
	AA (*n* = 6)	GA + GG (*n* = 26)		AA (*n* = 7)	GA + GG (*n* = 14)	
FBG (mmol/L)	−3.94 ± 6.25	−1.63 ± 1.95	0.015^a^	−1.56 ± 2.61	−1.81 ± 2.37	0.641
PBG (mmol/L)	−9.63 ± 5.83	−4.56 ± 5.84	0.067	−3.32 ± 6.46	−5.32 ± 5.79	0.310
FINS (mU/L)	5.17 ± 6.89	4.71 ± 4.28	0.252	3.25 ± 8.35	4.17 ± 6.41	0.865
PINS (mU/L)	−31.68 ± 56.56	−4.61 ± 20.91	0.041^a^	−13.72 ± 25.71	−14.93 ± 22.25	0.799
HbA1c (%)	−3.25 ± 3.11	−3.33 ± 6.30	0.487	−1.84 ± 1.43	−1.76 ± 2.13	0.778
TG (mmol/L)	−4.15 ± 6.97	−1.05 ± 2.70	0.061	−0.77 ± 2.20	0.69 ± 4.05	0.466
TChol (mmol/L)	−1.28 ± 1.56	−0.65 ± 1.46	0.197	−1.33 ± 1.15	0.32 ± 1.26	0.013^b^
LDL-C (mmol/L)	0.38 ± 1.35	−0.09 ± 0.76	0.148	−0.66 ± 0.49	−0.11 ± 1.38	0.228
HDL-C (mmol/L)	0.09 ± 0.25	−0.04 ± 0.20	0.134	−0.10 ± 0.15	−0.03 ± 0.11	0.205
HOMA-IR	−2.02 ± 7.50	1.22 ± 1.36	0.014^a^	0.34 ± 2.33	1.10 ± 2.77	0.841
HOMA-IS	−0.01 ± 0.02	−0.01 ± 0.02	0.440	−0.02 ± 0.08	0.003 ± 0.05	0.345
HOMA-B	1.11 ± 0.74	0.81 ± 1.57	0.441	1.62 ± 2.90	0.57 ± 0.86	0.149
QUICKI	−0.07 ± 0.13	−0.07 ± 0.12	0.447	−0.10 ± 0.42	0.0003 ± 0.25	0.437

BMI: body mass index; FBG: fasting blood glucose; PBG: postprandial blood glucose; FINS: fasting insulin; PINS: postprandial insulin; HbA1c: glycosylated haemoglobin; TG: triglycerides; TChol: total cholesterol; LDL-C: low-density lipoprotein cholesterol; HDL-C: high-density lipoprotein cholesterol; HOMA-IR: homeostasis model assessment of insulin resistance; HOMA-IS: homeostasis model assessment of insulin sensitivity; HOMA-B: homeostasis model assessment of *β*-cell function; QUICKI: quantitative insulin sensitivity check index.

^a^ *p* < 0.05 for comparison of AA genotype with GA/GG genotype of *SLC47A1* rs2289669 polymorphism in *SLC22A1* rs594709 AA genotyped T2DM patients, adjusted for age, sex, and BMI, in dominant model.

^b^ *p* < 0.05 for comparison of AA genotype with GA/GG genotype of *SLC47A1* rs2289669 polymorphism in *SLC22A1* rs594709 GA or GG genotyped T2DM patients, adjusted for age, sex, and BMI, in dominant model.
